# Crosstalk between proteins expression and lysine acetylation in response to patulin stress in *Rhodotorula mucilaginosa*

**DOI:** 10.1038/s41598-017-14078-5

**Published:** 2017-10-18

**Authors:** Xiangfeng Zheng, Qiya Yang, Lina Zhao, Maurice Tibiru Apaliya, Xiaoyun Zhang, Hongyin Zhang

**Affiliations:** School of Food and Biological Engineering, Jiangsu University, Zhenjiang, 212013 Jiangsu People’s Republic of China

## Abstract

The proteomic and lysine acetylation (Kac) changes, accompanying degradation of patulin in *Rhodotorula mucilaginosa* were analyzed using tandem mass tagging and N6-acetyllysine affinity enrichment followed by LC-MS/MS. Proteomic results showed that expression level of short-chain reductase protein and glutathione S-transferase involved in detoxification was significantly up-regulated. In addition, the expression levels of zinc-binding oxidoreductase and quinone oxidoreductase that are involved in antioxidant process, ABC transport and MFS transport responsible for chemical transport were activated when treated with patulin. The quantitative real time PCR (qRT-PCR) result also indicated these genes expression levels were increased when treated with patulin. Kac changes accompanying degradation of patulin in *R. mucilaginosa* were also observed. Totally, 130 Kac sites in 103 proteins were differentially expressed under patulin stress. The differentially up expressed modified proteins were mainly involved in tricarboxylic acid cycle and nuclear acid biosynthesis. The differentially down expressed Kac proteins were mainly classified to ribosome, oxidative phosphorylation, protein synthesis and defense to stress process. Our results suggest that patulin exposure prompt *R. mucilaginosa* to produce a series of actions to resist or degrade patulin, including Kac. In addition, the Kac information in *R. mucilaginosa* and Kac in response to patulin stress was firstly revealed.

## Introduction

Patulin is a mycotoxin produced by fungal species of *Penicillium*, *Aspergillus* and *Byssochlamys* spp which contaminate fruits and related products^1,2^. Patulin has been reported to suppress protein synthesis by inhibiting ATPase, RNA polymerase and aminoacyl-tRNA synthetase which disrupts the transcription, translation and amonao acid transport^3^. Prolong exposure to patulin can cause mutagenic^4,5^, carcinogenic^6^, immunotoxic^7^, neurotoxic^8^, genotoxic and teratogenic effects^9,10^. Thus, patulin poses a health risk to humans and live stocks.

Biocontrol agents (BCAs) have been studied to evaluate their ability to control patulin, especially yeast. Some of the BCAs have shown the ability to absorb patulin through their cell walls^11,12^. Besides, many yeasts *Rhodosporidium kratochvilovae* ^13^, *Pichia caribbica* ^14,15^ and *Rhodosporidium paludigenum* ^16^ have been proved to degrade patulin through enzymes catalytic process. For instance, Castoria, *et al*.^13^ reported that *R. kratochvilovae* degraded patulin to desoxypatulinic acid (DPA). The major product of DPA was reported to be the final step of patulin biosynthesis, so the reverse function enzyme of the enzyme responsible for the last step of patulin biosynthesis was assumed^17^. However, the enzymes responsible for this biosynthesis still remains unknown. In addition, *P. caribbica* and *R. paludigenum* were shown to degrade patulin by the production of intracellular enzymes^15,16^. *R. mucilaginosa* has also been reported to degrade patulin^18^. Up till date, the degradation products of patulin using antagonistic yeast have been identified, but the process and molecular mechanism(s) involved still remains unknown.

In human cells, patulin causes the accumulation of reactive oxygen species (ROS)^19^ through the combination with cellular glutathione (GSH) which is related to the generation of ROS. In yeast, patulin was reported to induce the accumulation of ROS^23^. ROS accumulation leads to cell death by inducing the signal pathway in the apoptotic process^20–22^. The action of patulin on *Saccharomyces cerevisiae* revealed that the synthesis of rRNA, tRNA and mRNA were inhibited by patulin^24^. Investigation of the mechanisms of patulin inhibition by yeast was characterized by analyzing the yeast transcriptome treated with patulin. The results indicated that patulin induced yeast gene expression profiles similar to gene expression patterns obtained after treatment with synthetic chemicals^25^.

Ianiri, *et al*.^26^ reported that patulin decreased the expression of genes involved in protein synthesis, modification, transport of ions, cell division and cell cycle, which reduced yeast growth. However, the yeast growth was only temporarily inhibited when *Schizosaccharomyces pombe* was exposed to high concentration of patulin. The cell growth was recovered by change of yeast cells size and chromatin structure, indicating that the *S. pombe* cells withstand the high concentration of patulin stress^27^. Moreover, patulin was found to activate protein degradation, especially proteasome activities, sulfur amino acid metabolism, and the defense system for oxidative stress^25^. Ianiri, *et al*.^26^ also reported that patulin treatment leads to the production of ROS and oxidative stress that result in the activation of stress response mechanisms controlled by transcription factors. The up regulated genes were involved in oxidation-reduction, transport processes, glutathione and thioredoxin systems which are responsible for the defense against patulin^26^. Chen *et al*. (2017) reported that a total of 30 differential proteins involved in 10 biological processes were identified, and more than two-thirds of the differential proteins were down-accumulated. Notably, the expression level of short-chain dehydrogenase (gi|190348612) was markedly induced by patulin^28^. Ianiri *et al*.^26^ found that genes encoding short or medium chain dehydrogenases were up-regulated under patulin stress in *Sporobolomyces* sp. However, there are also poorly in researching the patulin degradation mechanism.

Many organisms have been found to produce a number of post-translational modification (PTM) by acylation of lysine^29,30^, including acetylation^31^, malonylation^32^, propionylation^33^, butyrylation^33^, and succinylation^34^, which are crucial for functional regulations of many prokaryotic and eukaryotic proteins. Kac is one important types of PTM protein acylation and plays a key role in many cellular processes including gene transcription and cellular metabolism^35^. Kac is evolutionarily conserved from eukaryotes to prokaryotes^36^. Kac regulated the defense response to stress, such as stimuli stress, UV-induced Stress^37^. In addition, Kac targets protein complexes and co-regulates major cellular function, which may influence the biosynthesis of antibiotics in *Bacillus amyloliquefaciens* and chemistry metabolic^38^.

Zheng *et al*. (2016) reported that proteomics analyses revealed that patulin treatment increased the expression level of these proteins which were involved in metabolism and stress response processes in *P. caribbica* ^15^. Miura, *et al*.^39^ reported that patulin can inhibit the protein prenylation in mouse. The yeast *R. mucilaginosa* was reported to degrade patulin indicating that proteins were activated in defense against the patulin toxicity^18^. However, there was no comprehensive and systematic study on proteome and Kac changes involvement in defense against patulin stress in *R. mucilaginosa*.

In this study, the proteome and Kac changes accompanying degradation of mycotoxin patulin in *R. mucilaginosa* was analyzed. Proteins purported to be involved in patulin degradation or in reponse to patulin stress were analyzed. The Protein-protein interaction (PPI) network between differentially expressed Kac proteins were also investigated. This study provides the first comprehensive view of the lysine acetylome of *R. mucilaginosa* and the Kac involvement in patulin degradation.

## Results

### Quantification of *R. mucilaginosa* proteome change in response to patulin and verification of gene expression levels by qRT-PCR

**Figure 1:**
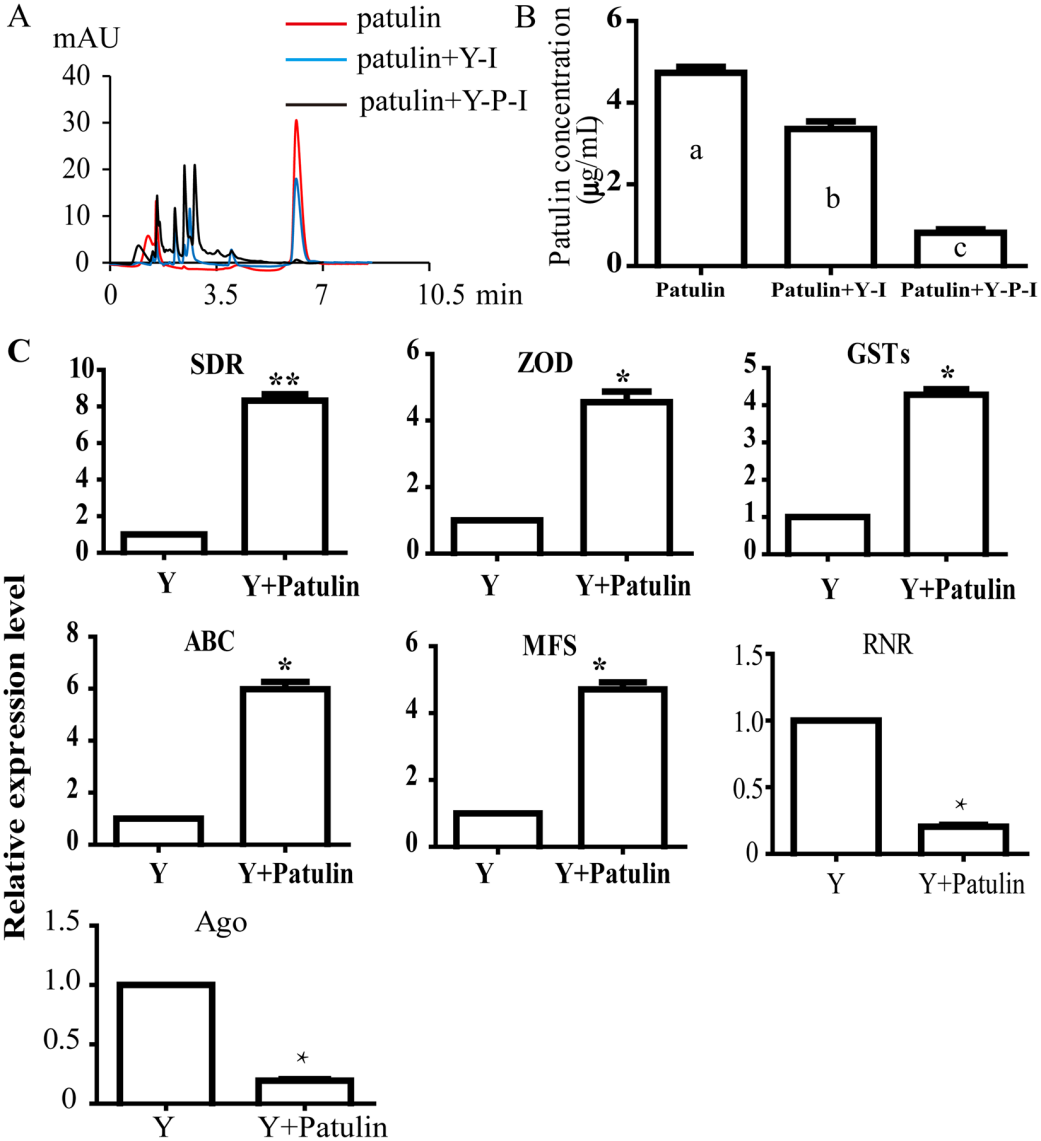
Patulin degradation effect of the intracellular enzymes of *R. mucilaginosa* and expression level of the genes involved in response to patulin stress. (**A**) Patulin degradation by intracellular enzymes of *R. mucilaginosa* (Y − I) and *R. mucilaginosa* amending with patulin(Y − P − I), the single peak before 7 min was the patulin. (**B**) Patulin content at 6 hpi. The data was conducted ‘one way anova’ analysis, different letter mean significant difference (*p* < 0.05). (**C**) The relative expression level of the genes which protein expression level was activated or inhibited. The data was analyzed by *t* test, *represent significant difference (*p* < 0.05) and **represent extremely significant difference (*p* < 0.01).

Patulin degradation by intracellular enzymes of *R. mucilaginosa* cells which was supplemented with patulin (Y − P − I) and the yeast without the patulin (Y − I) were analyzed at 6 hpi. As shown in Fig. 1A and B, the patulin content in the control was (4.73 μg/mL). However, the patulin content decreased to 3.36 μg/mL and 0.82 μg/mL when treated with Y − I and Y − P − I, respectively. The results indicated the patulin-induced intracellular enzymes that play important roles in degrading patulin in *R. mucilaginosa*.

The proteomes of Y (*R. mucilaginosa* alone) and Y + P (*R. mucilaginosa* treated with patulin) were analyzed by an iTRAQ-based proteomic analysis. In all, 2664 proteins were identified and 2243 were quantified. Out of these, 997 proteins were up-regulated and 1238 proteins were down-regulated, 8 proteins remained unchanged. Among them, 33 proteins were differentially up-regulated (DUR) (Y + PAT/Y Ratio > 1.5) and 25 proteins were differentially down-regulated (DDR) (Y + PAT/Y Ratio < 0.66). All the differentially up-regulated proteins (DUPs) are shown in Supplementary Table S2. The highest up-regulated protein was short-chain reductase (SDR), it increased 7.71 times in (Y − P − I). GSTs involved in detoxification was also significantly up-regulated. In addition, expression levels of 7 oxidoreductase and 9 dehydrogenase, which are responsible for maintaining the oxidation-reduction state of the metabolic substrate and reducing ROS stress, was DUR. The expression level of ABC transport (ABC) and MFS transport (MFS) were also increased when the patulin was added. Among the differentially down-regulated proteins (DDPs), Ribonucleoside-diphosphate reductase (RNR) which is involved in nuclear acid biosynthesis^40^ and some proteins which are involved in amino acid metabolism were DDP (Supplementary Table S3).

Expression level of the important genes which were up- or down-regulated were analyzed by qRT-PCR. As shown in Fig. 1C, the gene expression level of *SDR*, zinc-binding oxidoreductase (*ZOD*), *GSTs*, *MFS*, and *ABC* increased 8.32, 4.55, 4.28, 4.71 and 5.98 times in the *R. mucilaginosa* treated with patulin respectively compared to the control. In contrast, the expression level of *RNR* and Argonaute (*Ago*) were down-regulated by 4.89 and 5.12 times when treated with patulin.

### Bioinformatics analysis of the differentially expressed proteins in response to patulin stress

**Figure 2:**
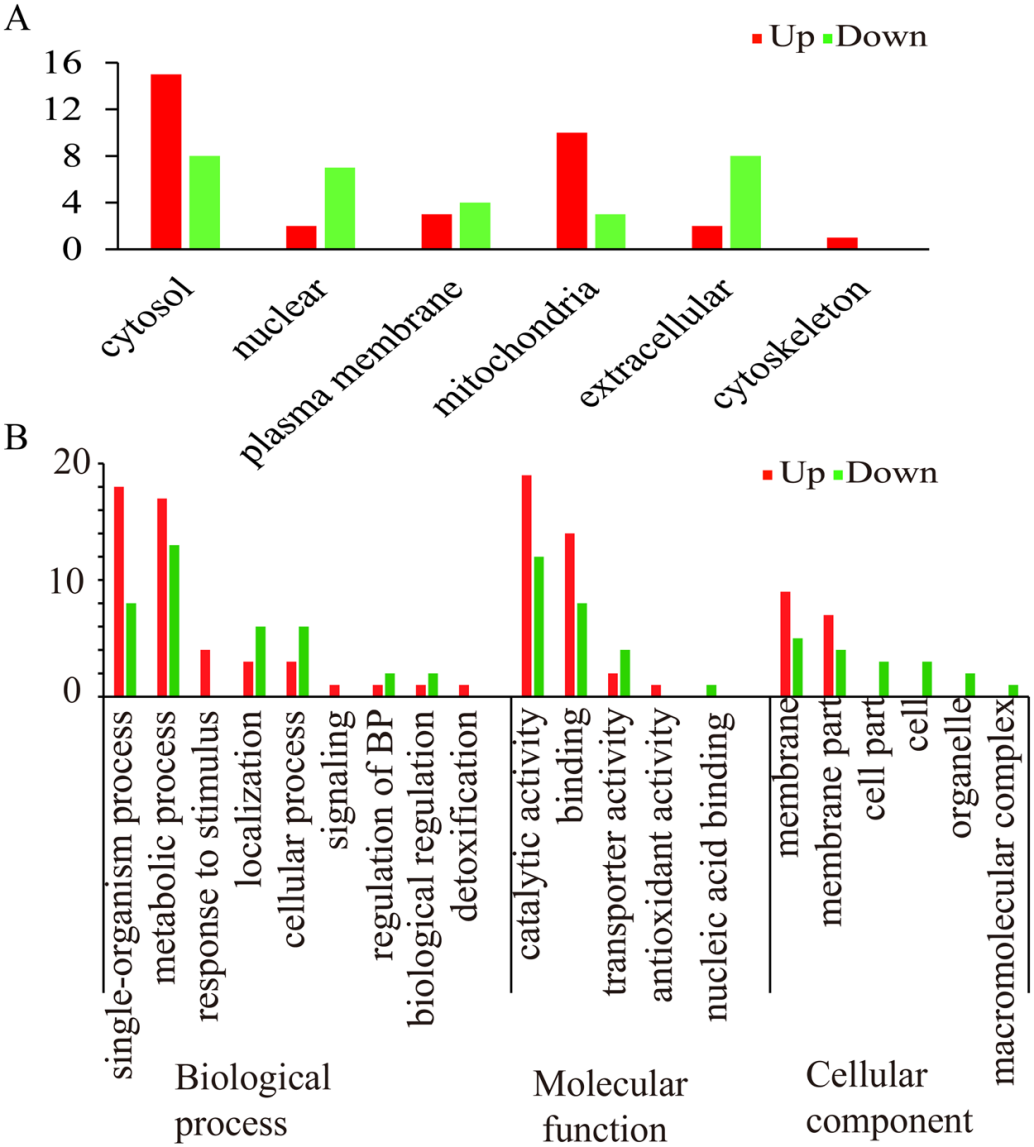
Subcellular localization and GO analysis of the proteins which were differentially expressed in response to patulin. (**A**) Subcellular localization of the differentially expressed proteins. (**B**) GO analysis of differentially expressed proteins. Red columns represent the differentially up regulated proteins, green columns represent the differentially down regulated proteins, Biological process (BP).

Subcellular localization prediction of the differentially expressed proteins (DEPs) showed that 15 DUPs were located in the cytosol and 10 DUPs were located in mitochondria, followed by plasma membrane (3), nuclear (2), extracellular (2) and cytoskeleton (1) (Fig. 2A). For the DDPs, they were observed to be distributed at cytosol (8), plasma membrane (8), mitochondria (7), nuclear (4) and extracellular (3) (Fig. 2A).

Gene ontology (GO) enrichment analysis was performed on all the DEPs. As shown in (Fig. 2B), most of the DUPs were classified under single-organism process (18) and metabolic process (17) of biological process. There were 4 proteins classified to response to stimulus and 1 proteins was classified to detoxification, which were responsible to the toxicity of patulin. Catalytic activity (19) and binding (14) category were the majority proteins classified under molecular function. Two proteins were classified to transporter activity and 1 protein was classified to antioxidant activity. Under the cellular component, 16 DUPs were all classified to membrane (9) and membrane part (7). Proteins involved in metabolic process (12) dominated the DDPs under the biological process, followed by single-organism process (7), localization (6) and cellular process (6). Catalytic activity (11) and binding (7) category also occupied the majority DDPs proteins under molecular function. Nine (9) DDPs were classified to membrane related and 6 DDPs were classified to cell part related component (Fig. 2B).

### Quantification and GO analysis of Kac proteins in response to patulin in *R. mucilaginosa*

MS data identified 707 acetylation sites in 342 proteins, of which 615 acetylation sites in 300 proteins were quantified. The modification level of acetylation sites was considered to be DUR if fold-change cutoff was greater than 1.2-fold, and DDP if less than 0.83-fold lower than control. The fold-change cutoff value means the value of normalized acetylation level (Kac (Y + PAT)/ Kac Y) with protein expression level (Y + PAT/Y). Based on this premise, 54 acetylation sites in 46 proteins were DUR (Supplementary Table S4) and 75 acetylation sites in 60 proteins were DDR (Supplementary Table S5) in response to stress caused by patulin. The acetylation level of pyruvate carboxylase, 2-oxoglutarate dehydrogenase, citrate synthase, isocitrate dehydrogenase, 2-oxoglutarate dehydrogenase E1 component, and 3-isopropylmalate dehydrogenase which were involved in TCA cycle were up-regulated due to the stress caused by the patulin. Besides, the acetylation level of phosphoglycerate kinase which is involved in glycolysis and transketolase which intend is involved in pentose phosphate pathway were up-regulated. In addition, the acetylation level of ribonucleoside-diphosphate reductase which was involved in catalysis the synthesis of deoxynucleotide were DUR (Supplementary Table S4). Among the DDR Kac proteins, 4 proteins were involved in oxidative redox reactions and defense response to ROS. They include zinc-binding oxidoreductase (ZOD), glutathione synthetase (GSHs), superoxide dismutase (SOD) and glutaredoxin (GRX). In addition, 11 modified sites in different ribosomal proteins, 5 modified sites of heat shock proteins and 3 modified sites of molecular chaperone proteins were differentially down-regulated (Supplementary Table S5).

**Figure 3:**
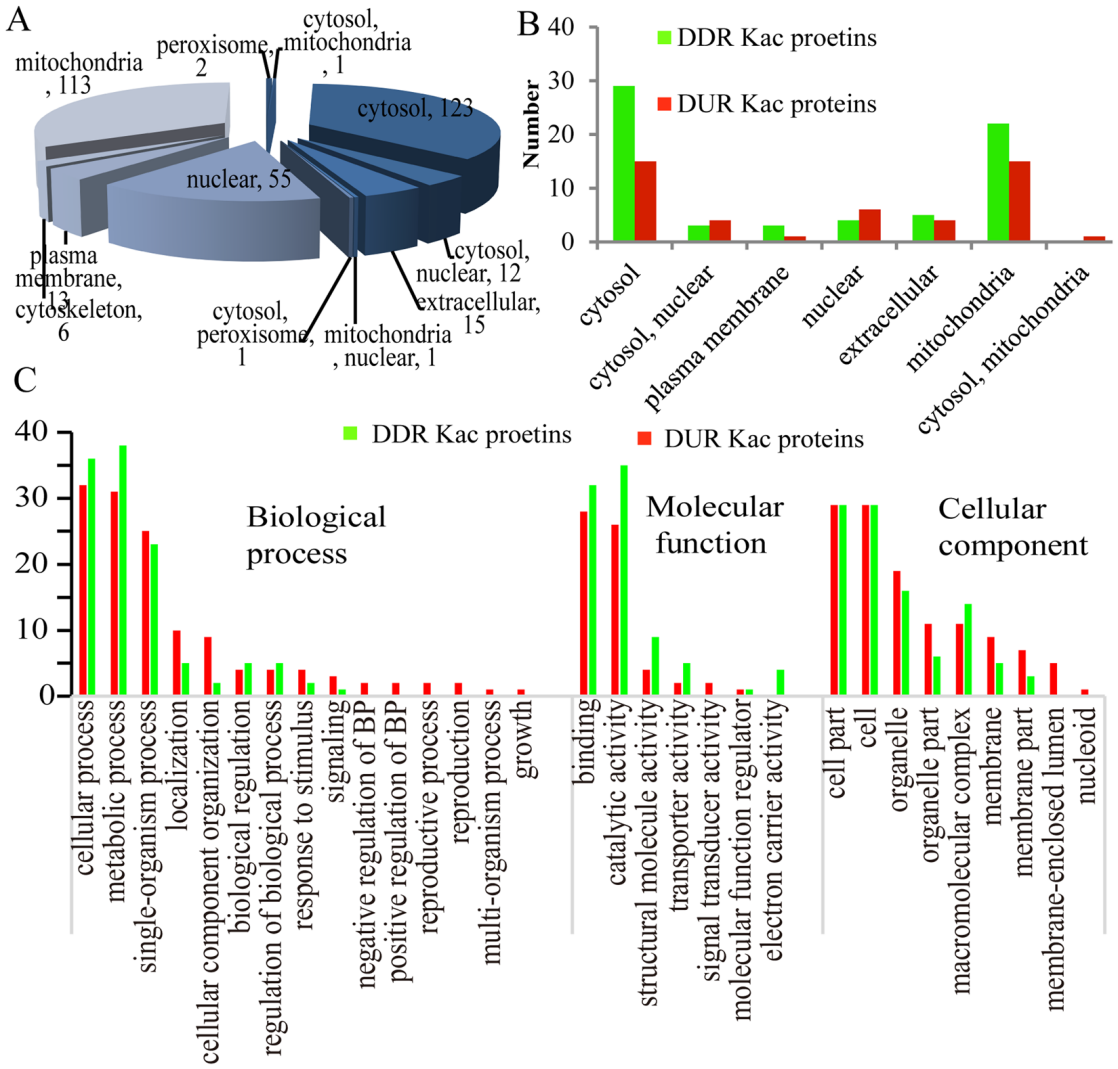
Subcellular localization and GO analysis of the Kac proteins which were differentially expressed in response to patulin. (**A**) Subcellular localization of all the Kac proteins in *R. mucilaginosa*. (**B**) Subcellular localization of the differentially expressed Kac proteins in reponse to patulin stress. (**C**) GO analysis of the differentially expressed Kac proteins in reponse to patulin stress. Red columns represent the differentially up regulated proteins, green columns represent the differentially down regulated proteins, Biological process (BP).

WoLF PSORT software was used to predict the subcellular localization of quantified proteins. As shown in Fig. 3A, 69% of all Kac proteins were distributed in the cytosol (123) and mitochondria (113). Most of the other proteins were distributed in the nucleus. As shown in Fig. 3B, fifteen cytosol proteins were DUR, and 29 were down-regulated. Meanwhile, 15 mitochondria Kac proteins, 6 nuclear Kac proteins, 4 cytosolic-nuclear Kac proteins and 4 extracellular Kac proteins were DUR, and 22, 4, 5 and 3 of these position proteins were DDR, respectively (Fig. 3B).

To better understand the Kac changes accompanying patulin degradation by *R. mucilaginosa*. The differentially expressed Kac proteins were performed GO analysis (2-level). Under the biological process, there are 32, 31, 25 DUR Kac proteins and 36, 38, 23 DDR Kac proteins were classified to cellular process, metabolic process and single organism process, respectively (Fig. 3C), followed by localization, cellular component organization or biogenesis, response to stimulus and regulation of biological process. Most of the differentially expressed Kac proteins were associated with binding and catalytic activity under the molecular function category. Under the cellular component category, most of the Kac proteins were classified to cell or cell part, organelle or organelle part and membrane or membrane part.

### Identification of Kac site motif and domain in response to patulin

**Figure 4:**
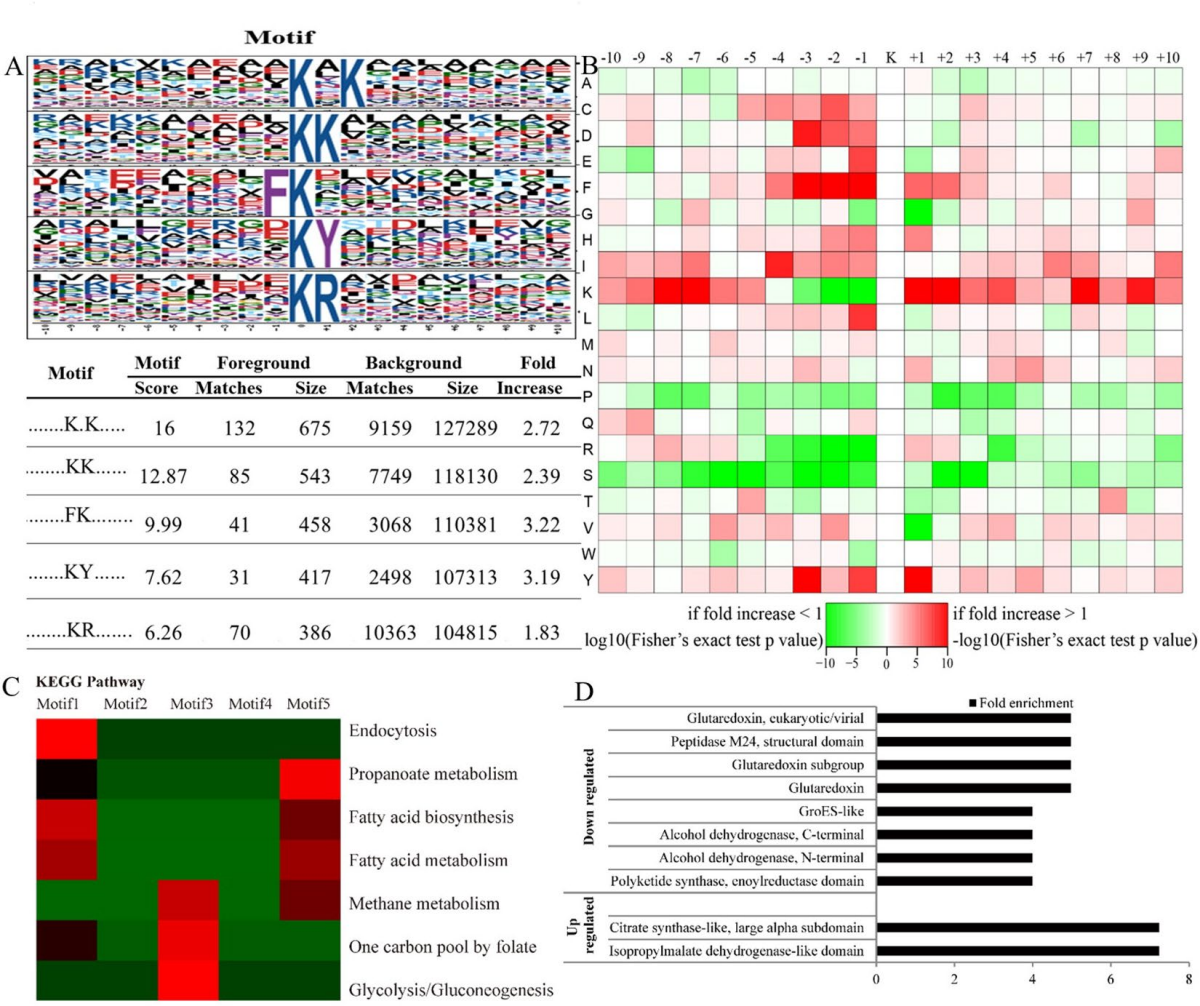
Bioinformational analysis of lysine acetylation sites. (**A**) Acetylation motifs and conservation of acetylation sites in response to patulin stress. Motif representation of significant motifs identified by Motif-X software. The motifs with significance of p < 0.000001 are shown. (**B**) Heat map of the amino acid compositions of the acetylation sites. (**C**) KEGG pathway analysis of the conservative Motif. (**D**) Domain enrichment analysis of the differentially expressed Kac proteins in response to patulin.

Kac site may be conservative in *R. mucilaginosa* in response to patulin. Thus, the consensus sequence motif around Kac sites was analyzed by motif-x software with acetyl-21-mer sequences. A total of five significantly enriched Kac sites motifs were identified. Lysine (K) was observed in the +1 and +2 position of two type motifs. Phenylalanine (F) was generally present in the −1 position, tyrosine (Y) and arginine (R) were observed in the +1 position (Fig. 4A). The lysine was particularly abundant at sites surrounding Kac sites. In addition, the isoleucine (I), phenylalanine (F), tyrosine (Y) and aspartic acid (D) were particularly abundant at sites surrounding Kac sites (Fig. 4B). These five motifs may play an important role in response to patulin stress. These five motifs were conducted Kyoto Encyclopedia of Genes and Genomes (KEGG) pathway analysis. The motif 1 was mainly involved in endocytosis, fatty acid biosynthesis and metabolism. Motif 3 was mainly involved in methane metabolism, one carbon pool by folate and glycolysis/gluconeogenesis. The motif 5 plays an important role in propanoate metabolism, fatty acid biosynthesis and metabolism and methane metabolism (Fig. 4C). The results indicated that regulating the metabolic processes by Kac is one of the ways to response patulin stress in *R. mucilaginosa*. The functions of proteins are often depending on their domains. Acetylation sites which were DUR are relatively abundant in isopropylmalate dehydrogenase-like domain and citrate synthase-like (Fig. 4D). DDR acetylation sites appeared to be more abundant associating with GRX protein, peptidase M24 structural domain, polyketide synthase domain, alcohol dehydrogenase, GroES-like, Rossmann-like and heat shock protein 70kD domain (Fig. 4D).

### Analysis of protein interaction networks of acetylated proteins in *R. mucilaginosa*

**Figure 5:**
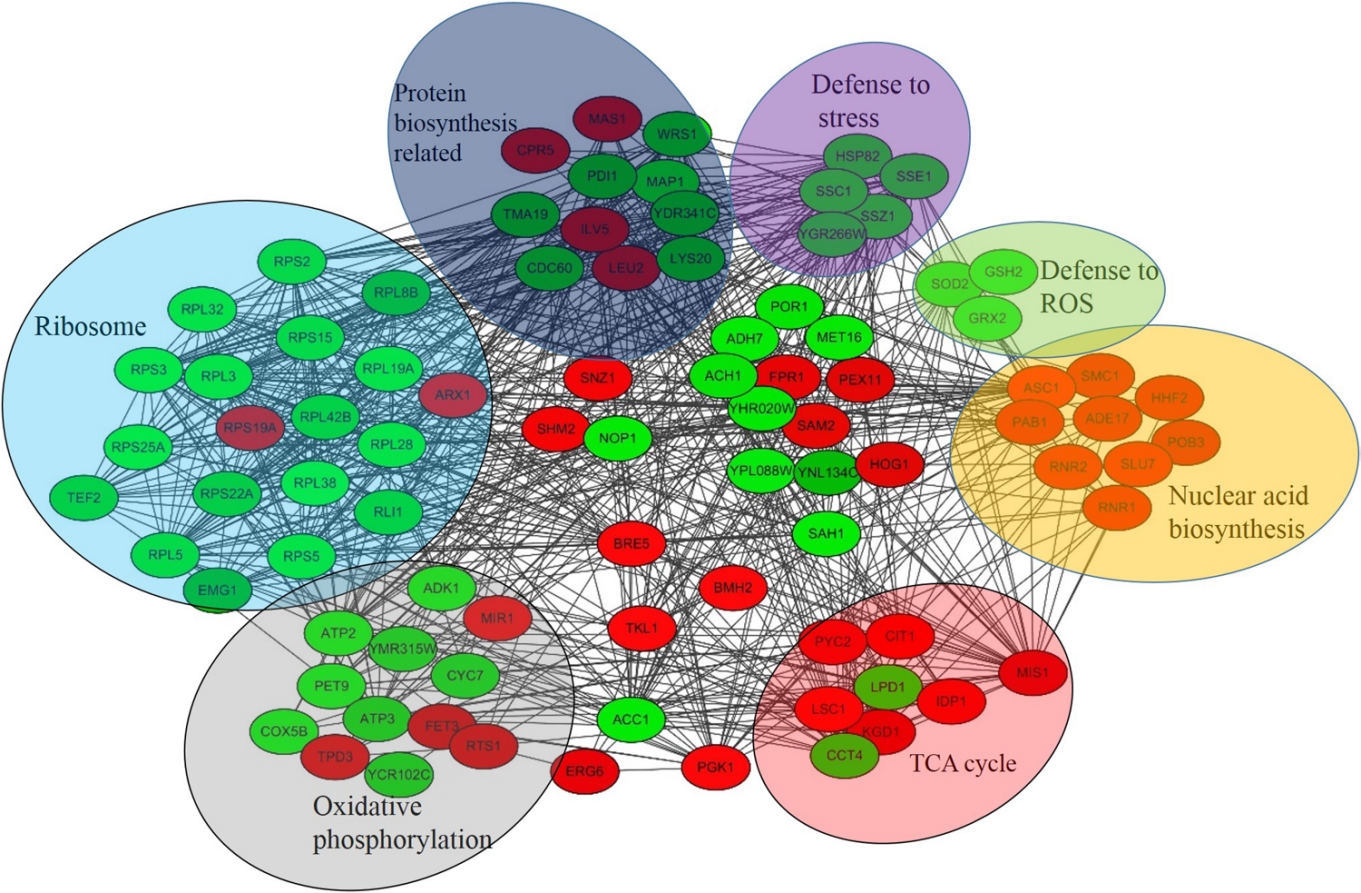
(**A**) Protein interaction network of differentially expressed Kac proteins. The Kac proteins were grouped using functional annotation and interaction network was visualized with Cytoscape. In the network figure, these proteins were divided into 2 categories that were colored differently: DUR protein (red); DDR protein (green). The black line represents the interaction relationship, the circle represents the different biological or metabolic process.

To further understand the cellular processes regulated by acetylation of *R. mucilaginosa* in response to patulin stress. The interaction network for all of the differentially expressed Kac proteins was determined using Cytoscape software with a confidence level of 0.7. In total, 88 lysine acetylated proteins are network nodes with different colors for DUR and DDR levels of acetylation proteins (Fig. 5), which presents a global view of how acetylated proteins perform various types of functions in *R. mucilaginosa*. The top 7 clusters identified include proteins associated with ribosome, oxidative phosphorylation, protein biosynthesis related proteins, TCA cycle, nuclear acid biosynthesis, defense to stress and ROS. However, the Kac level in these process was much differentially in response to patulin stress. Kac level of most of the Kac proteins of ribosome, protein biosynthesis related and oxidative phosphorylation was differentially down regulated. In addition, the Kac level of these proteins in response to stress or ROS was also differentially down-regulated. In contrast, the Kac level of proteins involved in TCA cycle and nuclear acid synthesis was DUR. The complicated interaction networks of acetylated proteins indicate that the interactions among these proteins complexes are likely to contribute to their cooperation and coordination in response to patulin stress.

## Discussion

Many yeasts have shown the capacity to withstand stress induced by patulin and degrade patulin. However, up to now, limited information on the molecular mechanism of the patulin degradation has been revealed. In this study, we investigated the global changes in proteome and Kac in *R. mucilaginosa* following treatment with patulin. Our results provide molecular mechanism of patulin degradation by *R. mucilaginosa*. In addition, we first reveal the proteins Kac information of *R. mucilaginosa* and the Kac information may be involved in patulin degradation.

Patulin combined with GSH led to ROS accumulation^41^. Patulin induce ROS accumulation, fluidization of the plasma membrane, change the chromatin structure in *Schizosaccharomyces pombe* ^27,42^. Suzuki and Iwahashi^43^ reported that SOD was activated by the patulin in *S. cerevisiae*. Ianiri, *et al*.^26^ reported the transcriptome change of *Sporobolomyces* sp. response to patulin and indicated that the up-regulated genes were those involved in oxidation-reduction and transport processes which resisted patulin toxicity and transported the mycotoxin out of the cells. In our result, the expression level of ZOD, NAD dependent oxidoreductase, quinone oxidoreductase and cytochrome-c peroxidase which are involved in oxidation-reduction process, ABC and MFS which were involved in transport processes were DUR both at gene and protein level. ABC and MFS transporter are the most important multidrug resistance transporters which usually transport chemical compounds out of the cell^44^. The result indicated that patulin exposure led to ROS accumulation in *R. mucilaginosa*, then genes involved in oxidation-reduction process and transport processes were activated to resist patulin toxicity and transport the mycotoxin out of the cells. Notably, compared with the control, the protein SDR was increased by 4.97 folds when treated with patulin. Expression level of corresponding gene was also significantly induced by patulin. The expression level of *SDR* was up-regulated under patulin stress in *Sporobolomyces* sp.^45^. Chen *et al*.^28^ also provided additional evidence to support the hypothesis that a short-chain dehydrogenase may be directly involved in the biodegradation of patulin in *Candida guilliermondii*. SDR plays critical roles in lipid, amino acid, carbohydrate, cofactor, hormone and xenobiotic metabolisms^46^. Our previous study found that patulin degradation maybe a series of steps^15^. Our study provided new evidence to support the hypothesis that *SDR* may be directly involved in one step of patulin degradation. In addition, patulin treatment resulted in an increment of expression level of GSTs, which catalyzes the conjugation of the reduced form of GSH to xenobiotic substrates for the purpose of detoxification or transfer the xenobiotic out of the cells. The result indicated GSTs may catalyzes the reduced form of GSH to patulin for the purpose of detoxifying the patulin. The function of SDR and GSTs in patulin degradation is ongoing.

PTMs have been reported to be associated with almost all known cellular pathways and disease process^30,31,34,47^. Kac is one of the PTMs that has been shown to be crucial in regulating protein functions^48^. Kac changes were found to regulate energy metabolism and the key metabolic pathways in diverse organisms in response to stress^49,50^. We conducted a standard detection of Kac in *R. mucilaginosa* treated with patulin. Interestingly, Kac level of ribosomal proteins, heat shock protein 70 family, ZOD, glutathione synthetase (GSHs), glutaredoxin (GRX), superoxide dismutase(SOD) were involved in ribosme and defense stress or ROS. But expression level of these proteins were almostly up regulated. Especially ZOD, the expression level was increased more than 3 times when treated with patulin, however the Kac level was DDR. Kac level of RNR was DUR, however expression level of it was differentially down-regulated. RNR was reported responsible for DNA biosynthesis^40^, and patulin was reported to inhibit DNA synthesis. Kurdistani and Grunstein^51^ reported that deacetylation is not only repressive but can be required for enzymes activity. As our result, the deacetylation of ZOD, GRX, SOD and GSHs may increase the activity of themselves to defense the ROS stress. Similarly, the increased acetylation of RNR may inhibits the activity of itself to prevent DNA synthesis. The result indicated that protein expression and Kac levels were both involved in response to patulin stress.

Provious study reported that 63% of mitochondrially localized proteins contain Kac sites^52,53^. In our findings, the Kac proteins were widely located at cytosol, mitochondria and nuclear where the main metabolic process exist. As Henriksen, *et al*.^54^ reported that the metabolic process located at cytosol, mitochondria and nuclear were more likely to be regulated by Kac in *S. cerevisiae*. The differentially expressed Kac proteins was also mainly distributed in the cytosol, mitochondria and nucleus of *R. mucilaginosa*. This demonstrated that these Kac proteins were involved in regulating the metabolic process of defense against the toxic of patulin and maintaining the growth of the yeast cells.

In *Candida albicans*, acetylation was reported to play significantly role in the regulation of metabolism^55^. The results indicated that Kac widely exist in *R. mucilaginosa* in regulating the main metabolic process. GO analysis of the differentially expressed proteins Kac proteins of *R. mucilaginosa* revealed that Kac proteins were abundant in catalytic activity and binding which are mainly involved in metabolic process, cellular process and single organism process that indicated Kac play an important role in response to patulin stress by regulating the important metabolic process.

Motif analysis according to the position of the residues and other properties of the residues around the acetylated lysine revealed three categories: the −1 position which is F, the +1 position which is (Y, R or K) and the +2 position which is (K). In *Candida albicans*, the Kac proteins motif were reported that at the +4 or +5 position is an alkaline residue with a long side chain (K or R) and the +1 or +2 position is a residue with a long side chain (Y, H, W or F)^48^. The result suggested the KY chain may be a conservative sequence of Kac in *R. mucilaginosa*. Domain analysis indicated that most of the down regulated domains were annotated to GRX. The GRXs are ubiquitous small heat-stable oxidoreductases that have proposed functions in many cellular processes^56^. It acts as general regulators of the redox state of disulfide groups in proteins, thus helping to defend against oxidative stress^57^. The DUR Kac domains were mainly annotated to the enzymes involved in the process of TCA cycle. The domain analysis result is corresponse with the result of DDR Kac proteins. Protein-protein interaction network (PPI) study suggested that Kac was observed having a variety of connections in oxidative phosphorylation and ribosome^54^. In our study, a variety of connections in ribosome, proteins biosynthesis related, oxidative phosphorylation, TCA cycle, nuclear acids synthesis, defense to stress and defense to ROS was observed, although Kac level of these proteins in these process differed. The result indicated that Kac plays an important role in regulating various metabolic processes in response to patulin in *R. mucilaginosa* and they mutually regulated each other. In addition, Kac changes was observed in many pathways from the PPI. The TCA cycle as a basic metabolic pathway in organism has been reported that the proteins Kac of TCA cycle play an essential role in response to UV-induced stress^37^. In this study, Kac level of Pyruvate carboxylase, Citrate synthase and Isocitrate dehydrogenase which were the key enzyme of TCA cycle were DUR by the stress caused by patulin. However, these proteins expression levels were not up-regulated by the patulin. The results suggested that the Kac regulated the TCA metabolic process in response to the stress induced by the patulin.

## Conclusion

**Figure 6:**
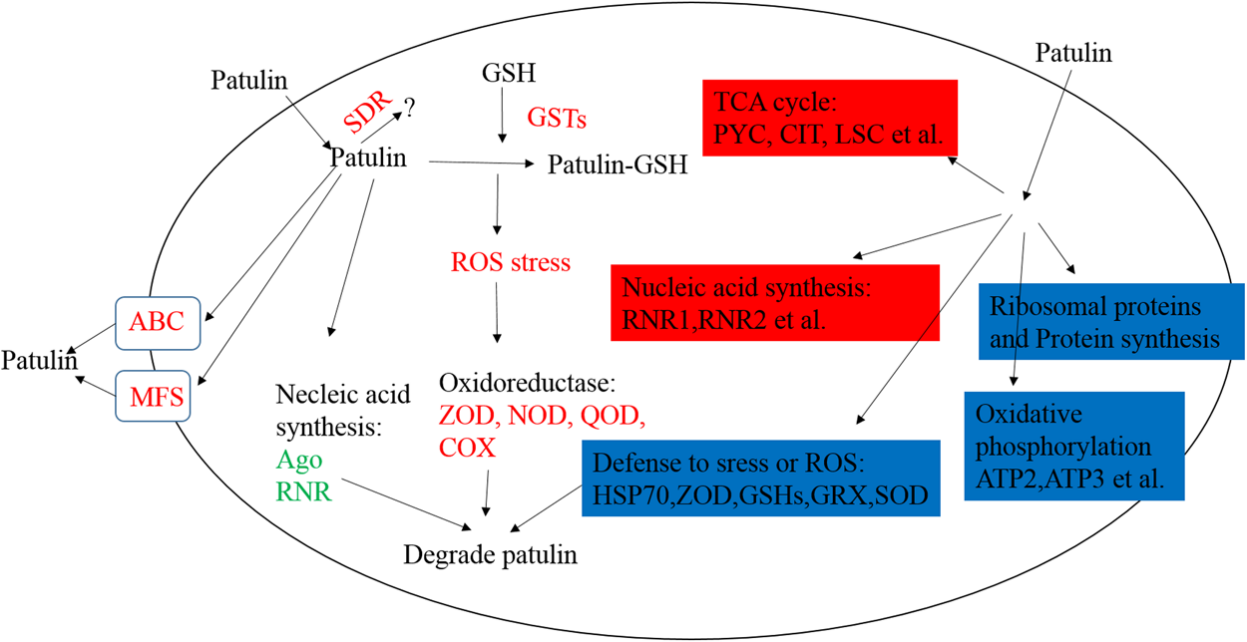
Global reponse of the *R. mucilaginosa* to the patulin stress. Red letters represent the proteins which were DUR, green letters represent the proteins which were DDR, red frames represent the Kac proteins which were DUR, and blue frames represent the Kac proteins which were DDR.

As shown in Fig. 6, patulin increase the ROS level and produce toxic to *R. mucilaginosa*, which induced yeast response to patulin stress which is critical for survival. ROS accumulation increased the expression levels of oxidoreductase to maintain the balance of redox state and to reduce the patulin toxicity in *R. mucilaginosa*. The ABC and MFS transporters were activated to efflux the toxin out yeast cells. In addition, proteins SDR, GSTs which are responsible for cleaning exogenous substances were activated to detoxify patulin. Protein acetylation is one of the potential regulatory mechanisms for rapid adaptation to patulin stress. Patulin treatment led to different Kac level of proteins in different process. The Kac level of nucleic acid synthesis related proteins was DUR. In addition, the enzymes involved in the key metabolic pathway of TCA cycle were also DUR. In contrast, the Kac level of oxidoreductase was DDR, although the expression level of these enzymes was DUR. In conclusion, crosstalk between proteins expression level and lysine acetylation level was observed in response to patulin stress in *R. mucilaginosa*.

## Materials and Methods

### Degradation of Patulin by Intracellular Enzymes

The intracellular enzymes were extracted as reported by Zheng *et al*. (2016) with a little modification. One milliliter of *R. mucilaginosa* (10^8^ cells/mL) was incubated in NYDB medium (0.8% nutrient broth, 0.5% yeast extract, 1% glucose) (Sangon Biotech, Shanghai, China)) or NYDB containing 5 µg/mL patulin in a rotary shaker at 180 rpm at 28 °C. At 24 hours post incubation (hpi), the cells were collected after centrifugation. The cells were washed with phosphate buffer (50 mM, pH 7.0) thrice. Three milligram wet yeast cells were quickly ground in mortar using pestle with liquid nitrogen added and then suspended in 10 mL phosphate buffer. After 30 min on ice (shock every 10 minutes), the samples were centrifuged at 13,000 × *g* for 10 min at 4 °C and the supernatant was collected. Afterwards, 25 µg of patulin was added to 5 mL of the intracellular enzymes, and 25 µg of patulin added to 5 mL of phosphate buffer as the control. Every treatment had three replicates and the experiment was replicated twice. At 6 hpi in a rotary shaker at 100 rpm at 28 °C, patulin was extracted by ethyl acetate and dissolved in water and CAN (9:1, v/v) used for HPLC analysis.

### HPLC-UV Analysis of Patulin

Agilent 1100 series system (Agilent, Santa Clara, CA, USA) was used to analysis the patulin as Zheng *et al*. (2016) reported. Briefly, the analytical column used was Zorbax, SB-C18 250 × 4.6 mm 5 µm (Agilent, Santa Clara, CA, USA). The mobile phase composed of water and CAN (9:1, v/v) that was set at 1 mL/min. The UV detection was performed at 276 nm. Data collection and subsequent processing was performed using Gilson Unipoint software 5.0 (Gilson, Inc, Middleton, WI, USA).

### Proteomics and Mass spectrometry sample preparation

Each three samples of Y+ patulin (yeast treated with patulin) and Y (yeast alone) were used to do proteomic analysis using tandem mass tagging (TMT) followed by LC-MS/MS. One milliliter of *R. mucilaginosa* (10^8^ cells/mL) was incubated in NYDB medium and NYDB containing 20 µg/mL patulin in a rotary shaker at 180 rpm at 28 °C. At 24 hours post incubation (hpi), the cells were collected by centrifuge and washed with phosphate buffer (50 mM, pH 7.0) thrice. The samples were quickly ground by adding liquid nitrogen, then the powdered cells were transferred to 5 mL centrifuge tube and sonicated three times on ice using a high intensity ultrasonic processor (Scientz, China) in lysis buffer (8 M urea, 2 mM EDTA, 3 μM TSA, 50 mM NAM 10 mM DTT and 1% Protease Inhibitor Cocktail). The mixture was separated by centrifugation at 20,000 g at 4 °C for 10 min. Finally, the protein was precipitated with cold 15% TCA for 2 h at −20 °C. After centrifugation at 4 °C for 10 min, the supernatant was discarded. The remaining precipitate was washed with cold acetone three times. The protein was dissolved in buffer (8 M urea, 100 mM TEAB, pH 8.0) and the protein concentration was determined with 2-D Quant kit according to the manufacturer’s instructions. The protein solution was reduced with 10 mM DTT for 1 h at 37 °C and alkylated with 20 mM IAA for 45 min at room temperature in darkness.

We performed a series of preliminary experiments using different amount of *R. mucilaginosa* proteins to find the best protocol. Following is the optimized experimental procedures based on these preliminary experiments. For trypsin digestion, the protein sample was diluted by adding 100 mM TEAB to urea concentration less than 2 M. Finally, protein was first digested using trypsin (1/50 protein mass) overnight following by digesting with trypsin (1/100 protein mass) for 4 h.

### TMT Labeling

After trypsin digestion, peptide was desalted by Strata X C18 SPE column (Phenomenex) and vacuum-dried. Peptide was reconstituted in 0.5 M TEAB and processed according to the manufacturer’s protocol for 6-plex TMT kit. Briefly, one unit of TMT reagent (defined as the amount of reagent required to label 1 mg of protein) was thawed and reconstituted in ACN. The peptide mixtures were then incubated for 2 h at room temperature and pooled, desalted and dried by vacuum centrifugation.

### HPLC Fractionation

The sample was then fractionated into fractions by high pH reverse-phase HPLC using Agilent 300Extend C18 column (5 μm particles, 4.6 mm ID, 250 mm length). Briefly, peptides were first separated with a gradient of 2% to 60% acetonitrile in 10 mM ammonium bicarbonate pH 10 over 80 min into 80 fractions, Then, the peptides were combined into 8 fractions and dried by vacuum centrifuging.

### Affinity Enrichment

Each one sample was used to do Kac analysis using Kac affinity enrichment followed LC-MS/MS. To enrich Kac peptides, tryptic peptides were dissolved in NETN buffer (100 mM NaCl, 1 mM EDTA, 50 mM Tris-HCl, 0.5% NP-40, pH 8.0), incubated with pre-washed antibody beads (PTM Biolabs) at 4 °C overnight with gentle shaking. The beads were washed four times with NETN buffer and twice with ddH_2_O. The bound peptides were eluted from the beads with 0.1% TFA. The eluted fractions were combined and vacuum-dried. The resulting peptides were cleaned with C18 ZipTips (Millipore) according to the manufacturer’s instructions, followed by LC-MS/MS analysis.

### Quantitative Proteomic Analysis by LC-MS/MS

Peptides were dissolved in 0.1% FA, directly loaded onto a reversed-phase pre-column (Acclaim PepMap 100, Thermo Scientific). Peptide separation was performed using a reversed-phase analytical column (Acclaim PepMap RSLC, Thermo Scientific). The gradient composed of 7% to 20% solvent B (0.1% FA in 98% ACN) for 35 min, 20% to 35% for 15 min and increased to 80% in 6 min then maintained at 80% for 4 min, at a constant flow rate of 300 nl/min on an EASY-nLC 1000 UPLC system, the resulting peptides was analyzed by Q ExactiveTM plus hybrid quadrupole-Orbitrap mass spectrometer (Thermo Fisher Scientific).

The peptides were subjected to NSI source followed by tandem mass spectrometry (MS/MS) in Q ExactiveTM plus (Thermo) coupled to UPLC. Intact peptides were detected in the Orbitrap at a resolution of 70,000. Peptides were selected for MS/MS using NCE setting as 30; ion fragments were detected in the Orbitrap at a resolution of 17,500. A data-dependent procedure that alternated between one MS scan followed by 20 MS/MS scans was applied for the top 20 precursor ions above a threshold ion count of 5E3 in the MS survey scan with 15.0 s dynamic exclusion. The electrospray voltage applied was 2.0 kV. Automatic gain control (AGC) was used to prevent overfilling of the orbitrap; 5E4 ions were accumulated for generation of MS/MS spectra. For MS scans, the m/z scan range was 350 to 1800. Fixed first mass was set as 100 m/z.

### Database Searching

The resulting MS/MS data was processed using MaxQuant with integrated Andromeda search engine (v.1.4.1.2). Tandem mass spectra were searched against protein database of *Rhodotorula toruloides/Rhodosporidium toruloides*, which were highly homologous with *R. mucilaginosa* and both are belonged to *Rhodosporidium* clade^58^. Trypsin/P was specified as cleavage enzyme allowing up to 4 missing cleavages, 5 modifications per peptide and 5 charges. Mass error was set to 10 ppm for precursor ions. Carbamidomethylation on Cys was specified as fixed modification and oxidation on Met, Acethylation on Lysine, and acetylation on protein N-terminal were specified as variable modifications. False discovery rate (FDR) thresholds for protein, peptide and modification site were specified at 1%. Minimum peptide length was set at 7. For quantification, TMT-6-plex was selected. All the other parameters in MaxQuant were set to default values. The site localization probability was set as >0.75. All the peptide sequences was deposited in the public protein peptide database MS-Viewer: http://msviewer.ucsf.edu/prospector/cgi-bin/msform.cgi?form=msviewer.

### Bioformatics analysis

Gene Ontology (GO) annotation proteome was derived from the UniProt-GOA database (www. http://www.ebi.ac.uk/GOA/). Firstly, Converting identified protein ID to UniProt ID and then mapping to GO IDs by protein ID. If some identified proteins were not annotated by UniProt-GOA database, the InterProScan soft would be used to annotated protein’s GO functional based on protein sequence alignment method by Blast2GO software. Then proteins were classified by Gene Ontology annotation based on biological process, molecular function and cellular component. Domain functional description was annotated by InterProScan based on protein sequence alignment method, and the InterPro domain database (http://www.ebi.ac.uk/interpro/) was used. We used Wolf PSORT to predict subcellular localization. Soft motif-x was used to analysis the sequences constituted with amino acids in specific positions of modify-21-mers (10 amino acids upstream and downstream of the site) in all protein sequences. And all the database protein sequences were used as background database parameter, other parameters with default.

To generate the PPI network, proteins that were selected from the all identified proteins were searched against the STRING database (v10.0) for protein-protein interactions. Only interactions between the proteins belonging to the searched data set were selected, thereby excluding external candidates. Interactions that have a STRING high confidence score (score ≥ 0.7, without “text-mining evidence”, see STRING instructions for details) were fetched. CytoScape (v3.3.0) was applied to visualize the interaction network generated from STRING.

### Verification of genes expression level by qRT-PCR

Each three RNA samples were extracted by the Yeast RNAiso Kit (Code No. 9751, TAKARA, Japan) according to the manufacturer’s instructions. The RNA concentration and purity was detected by Nanodrop2000 (Thermo scientific, USA). One μg of RNA was used to form cDNA by reverse transcription with PrimeScript™ RT reagent Kit with gDNA Eraser (Perfect Real Time) (Takara, Japan) followed manufacturer’s instructions. The primers were designed by Primer 5.0. All the primers are shown in supplementary Table S1. The qRT-PCR was conducted using an ABI 7300 Real-Time PCR System (Applied Biosystems, USA). The qRT-PCR system was 20 μl which contained 10 μl SYBR Premix Ex Taq II (Tli RNaseH Plus)(10×), each 0.8 μl Forward primer (10 μΜ) and Reverse primer (10 μΜ), 0.4 μl Reference Rox Dye (50×), 2 μl cDNA and distilled water up to 20 μl. The conditions were as follows: 95 °C for 30 s, 40 cycles of 95 °C for 5 s, 60 °C for 31 s, and a dissociation curve of 95 °Cfor 15 s, 60 °C for 1 min, 95 °C for 15 s followed the amplification cycle was added to determine the reliability of the quantitative results. Each sample have three technical replicates. Data were subjected to analysis of variance by using *t* test (SPSS release 17.0 for Windows; SPSS Inc., Chicago, Illinois, USA).

### Data availability

The datasets generated during and/or analysed during the current study are available from the corresponding author on reasonable request.

## Electronic supplementary material

Supplementary Table S1

Supplementary data S6

Dataset 1

Dataset 2

Dataset 3

Dataset 4
