# Author Correction: Crosstalk between proteins expression and lysine acetylation in response to patulin stress in *Rhodotorula mucilaginosa*

**DOI:** 10.1038/s41598-020-67059-6

**Published:** 2020-06-12

**Authors:** Xiangfeng Zheng, Qiya Yang, Lina Zhao, Maurice Tibiru Apaliya, Xiaoyun Zhang, Hongyin Zhang

**Affiliations:** School of Food and Biological Engineering, Jiangsu University, Zhenjiang, 212013 Jiangsu People’s Republic of China

Correction to: *Scientific Reports* 10.1038/s41598-017-14078-5, published online 18 October 2017

This Article contains errors in the Introduction.

“The major product of DPA was reported to be the final step of patulin biosynthesis, so the reverse function enzyme of the enzyme responsible for the last step of patulin biosynthesis was assumed ^17^.”

should read:

“Ascladiol was another major product of patulin degradation and also acts as the substrate for the last step of patulin biosynthesis. So, the enzyme responsible for the last step of patulin biosynthesis was also assumed to possess the reverse function and to be involved in patulin degradation ^17^.”

