# Supplementary material for: Crosstalk between proteins expression and lysine acetylation in response to patulin stress in Rhodotorula mucilaginosa
Source: Sci Rep. 2017 Oct 18;7:13490. doi: 10.1038/s41598-017-14078-5 (PMC5647337; doi:10.1038/s41598-017-14078-5)
Supplement: Supplementary file 1 — Supplementary Table S1 [file 41598_2017_14078_MOESM1_ESM.pdf]

Crosstalk between proteins expression and lysine acetylation in response to patulin stress in *Rhodotorula mucilaginosa*

Xiangfeng Zheng, Qiya Yang, Lina Zhao, Maurice Tibiru Apaliya, Xiaoyun Zhang, and Hongyin Zhang *

School of Food and Biological Engineering, Jiangsu University, Zhenjiang 212013, Jiangsu, People's Republic of China

*correspondence author: Hongyin Zhang

School of Food and Biological Engineering, Jiangsu University, 301 Xuefu Road, Zhenjiang, 212013, Jiangsu, People's Republic of China

E-mail addresses: zhanghongyin126@126.com

Tel.: +86-511-88780174; Fax: +86-511-88780201

Supplementary Table S1. The primers used for qRT-PCR.

QRT-SDR-F：	5' CGACAGCGTCTCGGATGA 3'
QRT-SDR-R：	5' GGGCGAGGGCTTTGTAGAT 3'
QRT-ZOD-F:	5' AAGGGCAACACCGAAGTCC 3'
QRT-ZOD-R:	5' AAGTCGCAACCCAACCAA 3'
QRT-MFS-F:	5' CGACGCAAACAAGGAGGC 3'
QRT-MFS-R:	5' CAATCAGAGCGGCGATAAAG 3'
QRT-GSTS-F:	5' GGGACTCGTCTTCCTCACC 3'
QRT-GSTS-R:	5' CATTCGGACTTCTGTTCATTTT 3'
QRT-ABC-F:	5' GGCGAAACCAAAGGGACT 3'
QRT-ABC-R:	5' CAAGCGACAACAGTAGGAAGG 3'
QRT-RNR-F:	5' GACCCGCAAGCAGCACAA 3'
QRT-RNA-R:	5' TCGTCGGCGTCGCAAGTA 3'
QRT-AGO-F:	5' AGTTCCGCTCGGTCATCC 3'
QRT-AGO-R:	5' TTGTAGGTGCGAAGGGTCTC 3'
QRT-ACT-F:	5' CGGGACATCAAGGAGAAGC 3'
QRT-ACT-R:	5' AGGAAGGAGGGCTGGAAGA 3'
