# Supplementary material for: Crosstalk between proteins expression and lysine acetylation in response to patulin stress in Rhodotorula mucilaginosa
Source: Sci Rep. 2017 Oct 18;7:13490. doi: 10.1038/s41598-017-14078-5 (PMC5647337; doi:10.1038/s41598-017-14078-5)
Supplement: Supplementary file 2 — Supplementary data S6 [file 41598_2017_14078_MOESM2_ESM.pdf]

Crosstalk between proteins expression and lysine acetylation in response to patulin stress in *Rhodotorula mucilaginosa*

Xiangfeng Zheng, Qiya Yang, Lina Zhao, Maurice Tibiru Apaliya, Xiaoyun Zhang, and Hongyin Zhang *

School of Food and Biological Engineering, Jiangsu University, Zhenjiang 212013, Jiangsu, People's Republic of China

*correspondence author: Hongyin Zhang

School of Food and Biological Engineering, Jiangsu University, 301 Xuefu Road, Zhenjiang, 212013, Jiangsu, People's Republic of China

E-mail addresses: zhanghongyin126@126.com

Tel.: +86-511-88780174; Fax: +86-511-88780201

Supplementary data S6. The search key and the URL of the database where our proteomic data deposit.

Kac proteins data:

http://msviewer.ucsf.edu/prospector/cgi-bin/msform.cgi?form=msviewer

Search key: wa8igxwcwy

Protiens peptides data:

http://msviewer.ucsf.edu/prospector/cgi-in/mssearch.cgi?report_title=MS-Viewer&search_key=x40u8jfys5&search_name=msviewer

Search key: x40u8jfys5
